# Accuracy of real-time shear wave elastography in staging hepatic fibrosis: a meta-analysis

**DOI:** 10.1186/s12880-020-0414-5

**Published:** 2020-02-11

**Authors:** Juan Fu, Biao Wu, Huazhi Wu, Feng Lin, Wei Deng

**Affiliations:** 1 Department of Infectious Disease, Hainan General Hospital, Haikou, China; 2 Department of Oral and Maxillofacial Surgery, Hainan General Hospital, 19 Xiuhua Road, Haikou, 570311 China

**Keywords:** Cirrhosis, Hepatic fibrosis, Elastography, Meta-analysis

## Abstract

**Background:**

Chronic liver disease (CLD) is an important cause of morbidity and mortality and can lead to hepatic fibrosis. This study was conducted to evaluate the diagnostic value of real-time shear wave elastography (SWE) in the assessment of hepatic fibrosis.

**Methods:**

A systematic search of databases was performed for publications on SWE during the period between 2010 and 2017. The identified studies were analyzed using Meta-disc 1.4 software to integrate and analyze the data.

**Results:**

Eleven studies comprising 1560 patients were included for analysis. The pooled sensitivity, specificity and diagnostic odds ratio were 0.85 (95% CI: 0.82–0.87), 0.79 (95% CI: 0.76–0.82) and 30.81 (95% CI: 16.55–57.34), respectively for patients with a Metavir-score of ≥ F2; 0.87 (95% CI: 0.84–0.91), 0.84 (95% CI: 0.82–0.87), 41.45 (95% CI:18.25–94.45), respectively for patients with ≥ F3; 0.88(95% CI: 0.83–0.91), 0.91 (95% CI: 0.89–0.92), 67.18 (95% CI:30.02–150.31), respectively for patients with ≥ F4. The areas under the receiver operating characteristic curve of the three groups were 0.9147, 0.9223 and 0.9520, respectively.

**Conclusions:**

Our work demonstrates that SWE is highly accurate for detecting and staging hepatic fibrosis.

## Background

Chronic liver disease (CLD) is an important cause of morbidity and mortality and can lead to hepatic fibrosis, cirrhosis, portal hypertension, and hepatocellular carcinoma. CLD is a major health burden in the United States and around the world. During the course of CLD, the death and inflammation of hepatocytes can lead to excessive deposition of extracellular matrix and abnormal distribution, resulting in hepatic fibrosis and its complications. If not treated timely, this would eventually develop into cirrhosis [1]. The causes of this condition include the infection with hepatitis viruses, compromised autoimmune response, poisoning and metabolic damage. Effective treatment methods for CLD are now available and can prevent progression of the hepatic fibrotic process or even result in regression of fibrosis when administered in the early stages of fibrosis [2]. Currently, percutaneous liver biopsy is the standard of reference for clinical assessment of hepatic fibrosis [3, 4]. However, it is invasive and may cause complications because of puncture, although the assessment is accurate [5]. A reliable noninvasive technique is needed for detecting and staging fibrosis as well for as evaluating treatment response [6]. In the recent years, several quantitative and noninvasive techniques have become available for measurements of liver stiffness, such as real-time tissue elastography (RTE), transient elastography (TE) (Fibroscan; Echosense, Paris, France), and real-time shear wave elastography (SWE) (Aixplorer, Supersonic Imagine, France) [7, 8]. Among them, SWE is a relatively new imaging technique that allows an estimation of the stiffness of the tissues in a quantitative way [9, 10]. It is based on the assumption that tissues that have pathological changes tend to be harder and less elastic than the surrounding healthy tissues. Estimation of the stiffness is based on the fact that the propagation of mechanical waves is greater in less elastic materials. The very quick data acquisition enables the assessment of tissue elasticity in real time as a colour map of stiffness superimposed over a grey-scaled B-mode. Putting a region of interest (ROI) in the area being investigated provides quantitative information about tissue elasticity in kilopascals or meter per second [11]. At present, SWE technology has been widely and successfully used in diagnosis of various diseases such as diseases in gland, vessel wall and superficial organ [12, 13]. It is also used to detection of portal hypertension in cirrhosis which can use to reflect the liver stiffness with comparable or better success rate and accuracy as compared with TE [14] and can be used to diagnose patients with or without clinical significant portal hypertension [15].

Although many studies, including meta-analysis [16], have shown that liver elasticity measured by SWE is related to the pathological stage of fibrosis, the accuracy and diagnostic thresholds based on SWE are still controversial and the quality criteria and optimal number of measurements need to be defined [17]. Therefore, we performed a meta-analysis to assess the overall performance of SWE in the diagnosis of liver fibrosis.

## Methods

This study was performed according to the Preferred Reporting Items for Systematic Reviews and Meta-Analyses statement [18]. Because this meta-analysis did not involve identifiable patient information, investigational review board approval was not necessary.

### Literature search

A systematic search of PubMed (MEDLINE), Embase, Scopus, the Cochrane Library, the Web of Science, Cumulative Index to Nursing and Allied Health Literature, Google Scholar, China knowledge Network (http://www.cnki.net/), China Biology Medicine disc (http://www.sinomed.ac.cn/zh/), VIP (http://qikan.cqvip.com) and Wanfangd (http://www.wanfangdata.com.cn) databases was performed for the 7-year period prior to May 2017. An initial search strategy involving the following free text words “hepatic fibrosis,” “elastography,” “liver cirrhosis,” “elasticity imaging techniques”, “fibroscan”, “liver physiology,” “liver stiffness,” “liver elasticity,” “elasticity imaging techniques/methods,” “ultrasonic elasticity imaging”, “sensitivity and specificity,” “reproducibility,” “repeatability,” and “reliability.” In addition, a manual search of reference lists from primary studies was performed to locate any potential studies missed with electronic search strategies. The identified studies were then screened independently by two observers to identify studies that enable analysis of diagnostic odds ratio.

### Inclusion and exclusion criteria

Inclusion criteria were as follows: *(a)* studies those study subjects were patients with liver fibrosis caused by various causes; *(b)* studies that used SWE to detect and stage the severity of fibrosis; *(c)* studies that clearly staged liver fibrosis based on the Metavir-score [19]. If 2 or more publications came from the same study, the publications with larger sample size were included. The exclusion criteria were as follows: *(a)* duplicate publication (based on the same primary study), *(b)* non-original research (such as reviews) and *(c)* studies with less than 30 patients. The final list of studies that met the inclusion and exclusion criteria were reviewed by all authors.

### Data extraction and quality verification

The data were extracted by using a predefined form. The following data were extracted: *(a)* author, journal, and year of publication; *(b)* number of subjects; *(c)* cause of disease; *(d)* average age and gender; *(e)* data related to disease staging by SWE and liver biopsy and *(f)* data to calculate the number of true positive, false positive, true negative and false negative. Data quality was assessed by using the Quality Assessment of Studies of Diagnostic Accuracy (QUADAS)-2 tool [20]. Disagreements were resolved by discussion between the investigators.

### Statistical analysis

Meta-DiSc 1.4 (http://www.hrc.es/investigacion/metadisc_en.htm), a software for meta-analysis of test accuracy was used to calculate the sensitivity, specificity, diagnostic odds ratio (DOR) and the 95% confidence interval (95% CI) and to analyze summary receiver operating characteristic curves (SROC) and the area under curve (AUC) [21, 22]. Forrest plots of sensitivities and specificities were constructed of the accuracy of SWE assessment of fibrosis. ROC plots of DOR was performed to determine threshold effect. χ2 –test and Cochrane-Q were used to determine the heterogeneity of DOR. If inconsistency (I^2^) ≥ 25%, and *P* < 0.05, DOR was considered heterogeneous, and random effect mode was chosen. Otherwise, fixed effect mode was chosen.

## Results

### Study search

**Fig. 1:**
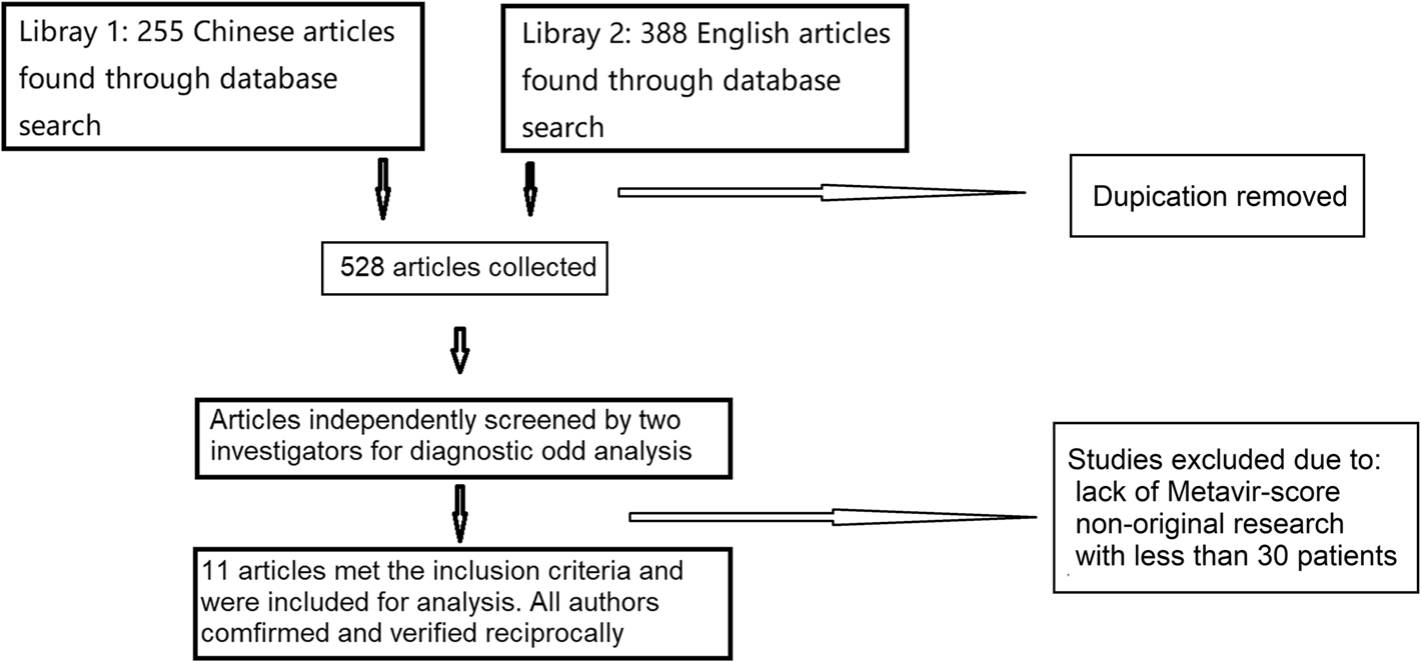
Flow diagram of studies included according to the inclusion and exclusion criteria

Two hundred and fifty-five Chinese paper and 388 English paper were revealed in the preliminary search. Duplicate articles were removed, and a list of 528 articles was collected in a single electronic library. After a detailed manual review, 11 articles met the inclusion and exclusion criteria. A flow diagram according to PRISMA guidelines of studies included is shown in Fig. 1. All studies included in the meta-analysis fulfilled four or more of the seven categories of the QUADAS-2 tool [20]. Baseline characteristics of included studies are shown in Table 1. The 11 articles which reported SWE-based fibrosis assessment were included for meta-analysis, were conducted in the United States, Italy, China, Korea, Japan, France, Romania and Turkey. There were 1560 patients aged between 12 to 82 years. The etiology of diseases was categorized as hepatitis C virus (HCV), hepatitis B virus (HBV), and others. The METAVIR scoring system was used to classify the disease severity, which was between F2 and F4. Among the studies, 11, 8 and 9 reported diagnostic value of SWE in patients with ≥ F2, ≥ F3 and ≥ F4, respectively. It should be mentioned that in the study conducted by Beland et al., the liver stiffness was measured in kPa on an ultrasound machine and converted to m/s by using a conversion formula [32] . The cut-off values of various etiologies are summarized in Table 2. Chronic viral liver diseases appeared to have lower cutoff values as compared with non-viral liver diseases such as non-alcoholic fatty liver disease, while autoimmune liver disease had high cut-off value (Table 2).

**Table 1:** Characteristics of studies assessing the performance of SWE for staging of liver fibrosis

Studies included	Year	Country	Averaged age (range)	Sample size	Etiology	Liver fibrosis stage
Ferraioli et al. [23]	2012	Italy	44.8 (19–76)	121	Chronic hepatitis C	F2, F3, F4
Jeong et al. [10]	2014	Korea	45.9 (12.0–82.0)	70	Chronic liver disease	F2, F3, F4
Samir et al. [9]	2015	US	47.0 (18–74)	136	Chronic liver disease	F2, F3, F4
Tutar et al. [24]	2014	Turkey	7.7 (0.3–17)	76	Chronic liver disease	F2
Tada et al. [25]	2015	Japan	61.0	55	Chronic hepatitis C	F2
Leung et al. [26]	2013	Korea	48.8	226	Chronic hepatitis B	F1, F2, F3, F4
Sporea et al. [27]	2014	Romania	52. 0 (18–82)	250	Chronic liver disease	F1, F2, F3, F4
Guibal et al. [28]	2015	France	54. 3	148	Chronic liver disease	F2, F3, F4
Zheng et al. [29]	2015	China	37. 7 (18–67)	167	Chronic liver disease	F2, F4
Yegin et al. [30]	2015	Turkey	45.3 (17–75)	105	Chronic liver disease	F1, F2, F3, F4
Zeng et al. [31]	2014	China	36.3 (20–59)	206	Chronic hepatitis C	F2, F3, F4

**Table 2:** The diagnostic threshold (kPa) of 2D-SWE in staging of liver fibrosis in patients with chronic liver disease

Etiology	≥F2	≥F3	F4	Studies
Chronic hepatitis C	7.1	8.7	10.4	Ferraioli et al. [23]
Chronic hepatitis C	7.3	8.9	9.6	Samir et al. [9]
Chronic hepatitis B	7.1	7.9	10.1	Leung et al. [26]
Chronic hepatitis B	7.2	9.1	11.7	Zeng et al. [31]
Chronic hepatitis B	8.8	11.5	18.1	Guibal et al. [28]
Nonalcoholic fatty liver disease	8.7	10.7	14.4	Cassinotto et al. [24]
Autoimmune liver disease	9.7	13.2	16.3	Zhang et al. [29]
Various chronic liver diseases	8.6	10.5	14.0	Jeong et al. [10]

### Meta-analysis

**Fig. 2:**
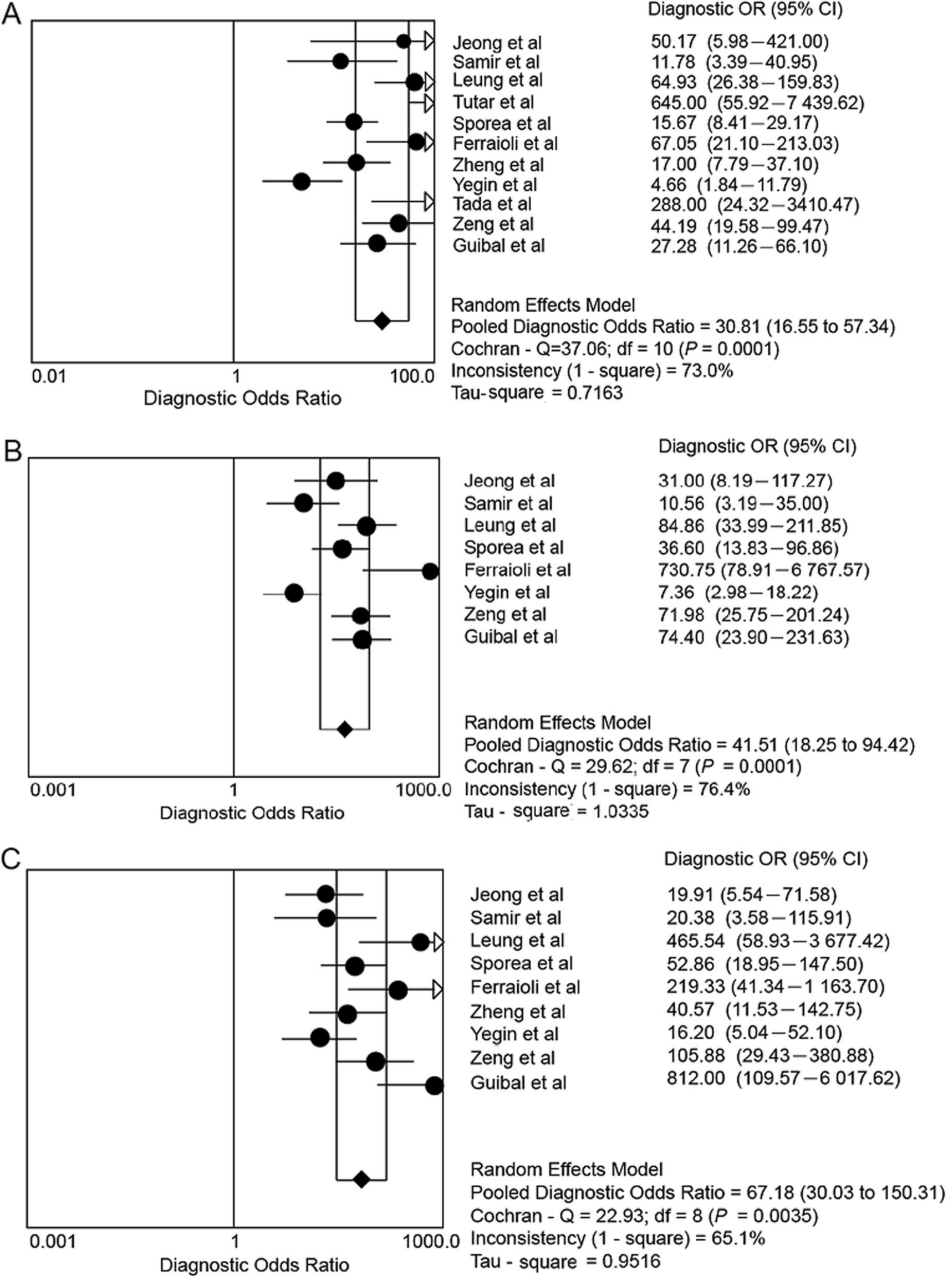
Forest plots of diagnostic odds ratios (DOR) from 11 test accuracy studies showing the results from 11 studies in detecting and staging hapetic fibrosis. **a** ≥ F2, **b** ≥ F3, and **c** ≥ F4

**Fig. 3:**
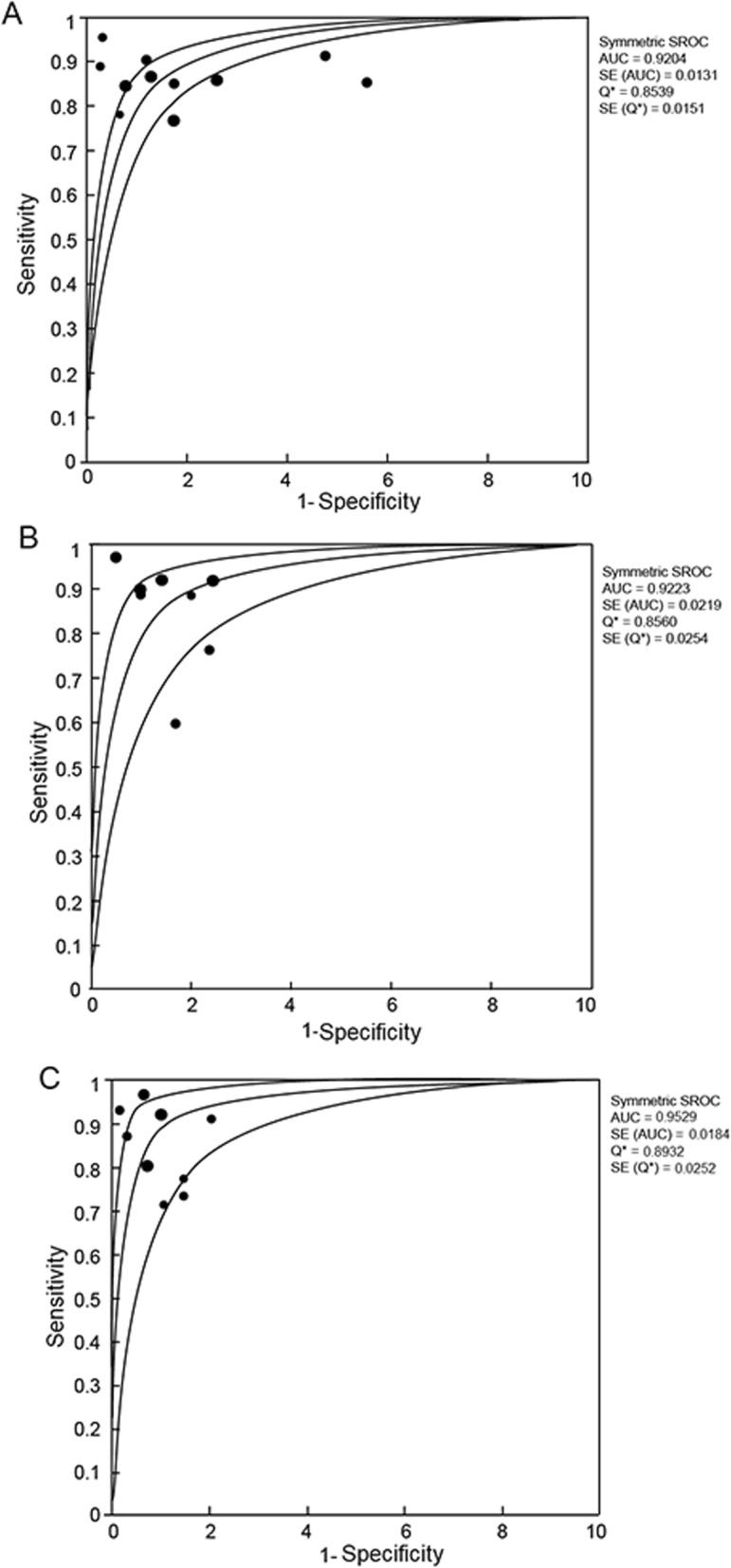
Summary receiver operating characteristic (SROC) curves with 95% confidenceintervals (shown as upper and lower curves) for estimating the diagnosis accuracy of SWE **a** ≥ F2, **b** ≥ F3, and **c** ≥ F4. Dots represent the sensitivity and specificity of each included study

Due to insufficient number of study (four out of 11 included studies) and number of patients (56 patients), studies that reported patients at the hepatic fibrosis stage of F0 to F1 were considered inappropriate for meta-analysis. I^2^ in ≥ F2, ≥ F3 and ≥ F4 groups were 73.0, 76.4 and 65.1% (Fig. 2), respectively, indicating that there were non-threshold effects in all three groups and could be analyzed by as random effect mode. The combined DOR of ≥ F2, ≥ F3 and ≥ F3 were 30.81 (95% CI: 16.55–57.34); 41.45 (95% CI: 18.25–94.45); and 67.18 (95% CI: 30.03–150.31) (Fig. 2). The sensitivity and specificity of DOR of ≥ F2, ≥ F3 and ≥ F3 groups were 0.85 (95% CI: 0.82–0.87) and 0.79 (95% CI: 0.76–0.82); 0.87 (95% CI: 0.84–0.91) and 0.84 (95% CI: 0.82–0.87); and 0.88 (95% CI: 0.83–0.91) and 0.91 (95% CI: 0.89–0.92), respectively. The AUC of DOR were 0.920, 0.922 and 0.952 in ≥ F2, ≥ F3 and ≥ F4 group, respectively (Fig. 3).

## Discussion

Our study shows SWE has high sensitivity and specificity to detecting and staging hepatic fibrosis in patients with ≥ F2 Metavir-score. The AUC of DOR in these patients are all over 0.92, suggesting that it has higher diagnostic values. There are several advantages of this method. Studies showed that it has excellent intraobserver and interobserver error, intraclass correlation coeffiients in three different sections of the liver ranged from 0.86 to 0.98, and the 95% confidence interval ranged from 0.71 to 0.99, suggesting the technique is highly reproducible [23, 26]. An non-invasive procedure, it is very suitable for detecting therapeutic outcome after operations, such as liver transplantation [33] and can be used for screening and subsequent management of liver diseases, particularly for children [34]. Other advantages of this method include determining therapeutic response, and monitoring age-related changes, including sarcopenia and clinical frailty syndrome. For example, the therapeutic outcome of Entecavir for chronic hepatitis B may be assessed using 2D-SWE to determine the change in stiffness of liver over a long term period to better treatment management and prognosis [35]. By quantifying mechanical and elastic tissue properties, SWE complements the diagnosis obtained at gray-scale (B-mode) US and power and color Doppler US [36].

The studies included in this analysis has high heterogeneity due to non-threshold effect. The main causes of heterogeneity were the etiology of liver fibrosis in the patients, which might result from infection of various hepatitis virus and fatty liver. Previous studies have shown that different causes of liver fibrosis may lead to different elastic threshold value for F2 or cirrhosis in TE-based assessment of fibrosis [37]. For liver fibrosis in chronic hepatitis C, the liver stiffness values were found to increase in parallel with degree of liver fibrosis when assessed with SWE and SWE was more accurate than TE in assessing significant fibrosis (≥ F2) [23]. SWE has the advantage of imaging liver stiffness in real time while guided by a B-mode image. Thus, the region of measurement can be guided with both anatomical and tissue stiffness information.

In this study, we analyzed 11 studies that met with inclusion criteria and found that the AUC was over 90% for fibrosis at F2, F3 and F4 stages, indicating that SWE is accurate to assess fibrosis at different stage.

The earliest technique FibroScan (FibroScan; Echosens, Paris, France) used in clinical assessment of liver elastic modulus is TE developed by Echosen. It is based on the measurement of the propagation velocity of shear waves in liver tissue and is noninvasive, quantitative and real-time technique to assess the degree of liver fibrosis. However, it is a one-dimensional imaging technology and needs special mechanical vibrating device to generate shear wave, and cannot be used for patients with ascites, obesity and stenosed rib space stenosis [38]. It cannot take two-dimensional images to avoid the non-target structure in the liver [7, 39]. SWE is a newer elastic imaging technology which is a newer variant of RTE [40]. Different from earlier technology, it uses shear-wave and does not need to compress and decompress the tissues to generate strain [41]. The shear waves are generated using acoustic radiation force automatically induced by the supersonic speed. This technique allows the measurement of the propagation speed of shear waves within tissues in meters per second (m/s) to locally quantify tissue stiffness (Young’s modulus) in kilopascals (KPa) and is less operator-dependent [42, 43]. 2D SWE is based on ultra-fast ultrasound tracking technology and Young’s modulus formula to display elastic images in real time and show the stiffness of the tissue with different color. It can avoid the inference from the structure of intrahepatic ducts to quantitatively assess the elastic modulus of liver tissue to quantify the stiffness of liver tissue hardness value, thus effectively increasing the accuracy of assessment and having broad clinical applications [44, 45]. On other side, RTE, also a noninvasive diagnostic technique that examines the stiffness and hardness of tissue, is mainly used to assess superficial tissues, such as neck, prostate, breast, and thyroid, by testing the elasticity [46, 47].

Our analysis showed that the pooled sensitivity (88%) and specificity (91%) of SWE in detecting and staging early cirrhosis (F1) are similar to those of TE [37, 48], acoustic radiation force impulse (ARFI) [49] and RTE [50, 51], those sensitivity and specificity are 83 and 89%, 87 and 87%, 74 and 84%, respectively. ARFI is very similar to SWE, which targets an anatomic region to be interrogated for elastic properties with a Region-of-Interest (ROI) cursor [52]. However, for significant fibrosis (F ≥ 2), the overall sensitivity and specificity of SWE are greater than these of TE and RTE, and similar to these of ARF. For TE and RTE, the combined sensitivity is 79% and the combined specificity is 78 and 76%, respectively; while for ARFI [49], the combined sensitivity and specificity are 74 and 83%. Clinically, the differentiation of nonadvanced (F0 and F1) and advanced (F2–F4) fibrosis is particularly relevant in HCV hepatitis C virus, where advanced fibrosis at the time of diagnosis has been shown to correlate with long-term cirrhosis risk, because once diagnosed as significant hepatic fibrosis (F ≥ 2), these patients have high risks to develop cirrhosis [53–55]. In this aspect, SWE may fill an important gap [56]. In addition, ultrasonic elasticity technologies, such as ARFI and TE, have some limitations and their measurements are affected by tissue inflammation and steatosis, which do not affect the SWE assessment of liver fibrosis [9]. For example, TE is adequate for a diagnosis of cirrhosis, but its accuracy for milder stages of fibrosis is much less satisfactory. ARFI was not associated with alanine aminotransferase (ALT), body mass index, Metavir grade, and liver steatosis. On other hand, TE was significantly correlated with the ALT value [57], but the accuracy of ARFI was influenced by sex, interquartile range interval, high alanine aminotransferases and high aspartate aminotransferases levels [58].

There are a number of factors that would affect the evaluation performance for the liver stiffness by SWE, such as the number of measurements, liver volumes, patient’s conditions such as overweight or obesity or other complications as well as the fibrosis stage and experience of operators [25, 27]. It is generally agreed that three measurements are sufficient to obtain consistent results for assessing liver fibrosis [11]. However, more measurements help reducing unreliable measurements [10]. The fibrosis stage, GGT and serum albumin are shown to significantly influence the stiffness measurement [31], although use of different equipment and working methodologies in different studies make it difficult to compare these influences [30]. The causes of chronic liver disease are also important factor that influence the liver stiffness. For example, the cutoff points for ≥ F1 in patients with chronic nonviral hepatitis (alcoholic or non-alcoholic steatohepatitis) and chronic viral hepatitis (B) were 6.5 kPa and 6.8 kPa, respectively [25, 27]. On other hand, as shown in Table 2, fibrosis etiologies such as HCV or HBV infections give similar SWE performances, while nonalcoholic fatty liver disease and autoimmune liver disease generate high liver stiffness. Although included studies did not report the influence of liver viscosity on the results of SWE, Deffieux et al. reported a correlated between viscosity and the degree of liver fibrosis, but not with steatosis or disease activity [59]. In addition, older age and higher BMI were associated with impossibility to obtain reliable measurements [25]. As such, quality criteria for 2D-SWE is very important to obtain reliable measurement. For example, Yoon et al. used a value of less than 0.3 for standard deviation/mean 2D-SWE value to control the measurement quality [60]. Age appears an important factors affecting the measurement and children are found to have higher cutoff value for liver fibrosis [24]. This, however, may be due to the difference in the etiology of chronic liver disease between children and adult population.

The cutoff values for the diagnosis of fibrosis were reported in some of the included studies (Table 2). Although the values increase generally with increased fibrosis stage, they are slightly different among the studies even the etiology is the same. For example, Zeng et al. reported that cutoff (kPa) for F2. F3 and F4 fibrosis stage were 7.2, 9.1 and 11.7 for chronic hepatitis B-induced liver fibrosis [31], while Guibal et al. found that these values were 8.8, 11.5 and 18.1 [28]. For children, the cut-off value could be even higher (10.6 kPa) [30]. It is likely that the machines and methodologies used in SWE would impact the cut-off value, as well as the site of measurement [9]. Therefore, it is important to develop institute-specific standard for SWE-based diagnosis for liver fibrosis.

Limitations of our study included inhomogeneity owing to causes of disease, small numbers of studies included. Studies with F0-F1 were not included due to insufficient data availability. In addition, not all subjects underwent biochemical hepatic tests and viral marker assessments for verification of etiology.

## Conclusion

Our study has shown that SWE is accurate in diagnosing significant, advanced fibrosis and early cirrhosis of the liver. However, since liver fibrosis has various etiology which may generate different liver stiffness as discussed above, the diagnosis cutoff threshold need to be adjusted case by case based on other information, such as laboratory assessment of the cause of the disease. Due to the limited number of studies included in this study, there is a need to further investigate the relationship between tissue elasticity and liver fibrosis severity.

## Data Availability

The datasets used and/or analysed during the current study are available from the corresponding author on reasonable request.

## Abbreviations

AUC: Area under curve
CI: Confidence interval
CLD: Chronic liver disease
DOR: Diagnostic odds ratio
HBV: Hepatitis B virus
HCV: Hepatitis C virus
QUADAS: Quality Assessment of Studies of Diagnostic Accuracy
ROI: Region of interest
RTE: Real-time tissue elastography
SROC: Summary receiver operating characteristic curves
SWE: Shear wave elastography
TE: Transient elastography
